# Proton stopping measurements at low velocity in warm dense carbon

**DOI:** 10.1038/s41467-022-30472-8

**Published:** 2022-05-24

**Authors:** S. Malko, W. Cayzac, V. Ospina-Bohórquez, K. Bhutwala, M. Bailly-Grandvaux, C. McGuffey, R. Fedosejevs, X. Vaisseau, An. Tauschwitz, J. I. Apiñaniz, D. De Luis Blanco, G. Gatti, M. Huault, J. A. Perez Hernandez, S. X. Hu, A. J. White, L. A. Collins, K. Nichols, P. Neumayer, G. Faussurier, J. Vorberger, G. Prestopino, C. Verona, J. J. Santos, D. Batani, F. N. Beg, L. Roso, L. Volpe

**Affiliations:** 1 Centro de Laseres Pulsados (CLPU), Parque Cientifico, E-37185 Villamayor, Salamanca Spain; 2 Princeton Plasma Physics Laboratory, 100 Stellarator Road, Princeton, NJ 08536 USA; 3 CEA, DAM, DIF, F-91297 Arpajon, France; 4 University of Bordeaux, CNRS, CEA, CELIA (Centre Lasers Intenses et Applications), UMR 5107, F-33405 Talence, France; 5 University of Salamanca, Salamanca, Spain; 6 Center for Energy Research, University of California San Diego, La Jolla, CA 92093 USA; 7 General Atomics, San Diego, CA 92121 USA; 8 University of Alberta, Department of Electrical and Computing Engineering. Edmonton, Alberta, T6G 2V4 Canada; 9 Goethe-Universität Frankfurt am Main, Max-von-Laue-Strasse 1, 60438 Frankfurt am Main, Germany; 10 Laboratory for Laser Energetics, University of Rochester, 250 E. River Road, Rochester, NY 14623 USA; 11 Theoretical Division, Los Alamos National Laboratory, Los Alamos, NM 87545 USA; 12 GSI Helmholtzzentrum für Schwerionenforschung GmbH, Planckstrasse 1, 64291 Darmstadt, Germany; 13 Université Paris-Saclay, CEA, LMCE, F-91680 Bruyères-le-Châtel, France; 14 Institute of Radiation Physics, Helmholtz-Zentrum Dresden-Rossendorf, Bautzner Landstrasse 400, 01328 Dresden, Germany; 15 Dipartimento di Ingegneria Industriale, Universitá di Roma “Tor Vergata”, Via del Politecnico 1, 00133 Roma, Italy; 16 Laser-Plasma Chair at the University of Salamanca, Salamanca, Spain; 17 Instituto Universitario de Física Fundamental y Matemáticas, 37008 Salamanca, Spain

**Keywords:** Laser-produced plasmas, Plasma-based accelerators

## Abstract

Ion stopping in warm dense matter is a process of fundamental importance for the understanding of the properties of dense plasmas, the realization and the interpretation of experiments involving ion-beam-heated warm dense matter samples, and for inertial confinement fusion research. The theoretical description of the ion stopping power in warm dense matter is difficult notably due to electron coupling and degeneracy, and measurements are still largely missing. In particular, the low-velocity stopping range, that features the largest modelling uncertainties, remains virtually unexplored. Here, we report proton energy-loss measurements in warm dense plasma at unprecedented low projectile velocities. Our energy-loss data, combined with a precise target characterization based on plasma-emission measurements using two independent spectroscopy diagnostics, demonstrate a significant deviation of the stopping power from classical models in this regime. In particular, we show that our results are in closest agreement with recent first-principles simulations based on time-dependent density functional theory.

## Abstract (web summary)

Charged particle interaction and energy dissipation in plasma is fundamentally interesting. Here the authors study proton stopping in laser-produced plasma for the moderate to strong coupling with electrons.

## Introduction

Ion stopping in warm dense matter (WDM) is an important topic in inertial confinement fusion (ICF) for the ignition of small-margin ICF targets by *α*-particle self-heating^1,2^ and for ICF schemes using ion beams as the main driver, like heavy-ion fusion^3,4^ or ion-driven fast ignition^5,6^. A precise knowledge of ion stopping in WDM is also essential for understanding proton transport in matter^7,8^ and for experiments where dense plasma states are generated using ion beams^9^, in particular proton isochoric heating^10,11^. Such experiments have applications for studying the structure^12^, the equation-of-state^13^ and the transport properties of dense plasmas^14^, like the conductivity^13,15^ and the thermal equilibration^16^ of WDM samples. Other applications include plasma diagnostics using ion beams^17,18^.

The WDM state is characterized by densities in the order or higher than the one of the solid state and temperatures below 100 eV. In this parameter range, the plasma is usually partially ionized as well as electron-coupled and -degenerate. These quantities are respectively measured by the non-dimensional parameters for electron coupling Γ and electron degeneracy Θ, whose values for the reached conditions are approximately

1 $${{\Gamma }}=\frac{{e}^{2}}{{a}_{{{{{{{{\rm{e}}}}}}}}}{k}_{{{{{{{{\rm{B}}}}}}}}}{T}_{{{{{{{{\rm{e}}}}}}}}}}\;\ge\; 0.1\quad \quad \,{{\mbox{and}}}\,\quad \quad {{\Theta }}=\frac{{k}_{{{{{{{{\rm{B}}}}}}}}}{T}_{{{{{{{{\rm{e}}}}}}}}}}{{E}_{{{{{{{{\rm{F}}}}}}}}}}\;\le\; 10,$$

where ${a}_{{{{{{{{\rm{e}}}}}}}}}={\left(4\pi {n}_{{{{{{{{\rm{e}}}}}}}}}/3\right)}^{-\frac{1}{3}}$ is the average distance between the electrons, and *E*_F_ is the Fermi energy of the free electron gas in the target. Electron coupling and degeneracy influence the electron distribution function and the plasma screening properties. This modifies the Coulomb logarithm characterizing the collisions in the plasma and, thus, the plasma transport quantities including the ion stopping power *dE*/*dx*.

Most ion-stopping experiments have been performed in classical, highly ionized plasmas, in ideal (Γ ≪ 1) and nondegenerate (Θ ≫ 1) conditions. Even in classical plasmas, measurements have chiefly been acquired at projectile velocities *v*_p_ much larger than the thermal velocity of the plasma electrons ${v}_{{{{{{{{\rm{th}}}}}}}}}=\sqrt{(3{k}_{{{{{{{{\rm{B}}}}}}}}}{T}_{{{{{{{{\rm{e}}}}}}}}}/{m}_{{{{{{{{\rm{e}}}}}}}}})}$ (*v*_p_ ≫ *v*_th_). In this high-velocity range, models are well-established and agree with experimental data^19–22^. In contrast, the parameter region where *v*_p_ ~ *v*_th_ (Bragg peak) is theoretically more challenging. The beam-plasma coupling is here determined by binary collisions as well as interactions with density waves. Their relative and absolute contributions are strongly temperature dependent, so that even for ideal or nondegenerate conditions large discrepancies between the predictions of different stopping-power models are reported^23,24^. Experiments probing the Bragg peak are also more challenging, and the few measurements carried out for classical plasmas support models that include close binary collisions in the beam-plasma interaction description^25,26^.

For WDM target conditions, theoretical modelling is more difficult due to electron coupling and degeneracy, and requires more advanced theories like quantum many-body approaches and first-principles calculations. This leads to even larger theoretical discrepancies than in classical plasmas, which increase for low projectile velocities and culminate near the Bragg peak. At low velocities, temperature and/or degeneracy effects are expected to be important on the stopping power and significant deviations from classical theories are predicted^27–31^.

Measurements in WDM are also more challenging because of shorter sample lifetimes and a more difficult target characterization due to high plasma densities. A few indirect stopping measurements in degenerate conditions have been extracted from ICF implosions by using tertiary neutron spectra^32,33^, but these data do not allow a precise benchmarking of stopping models. The only direct ion-stopping measurements in WDM reported so far have been performed at the OMEGA laser facility^34^. Projectiles were quasi-monoenergetic protons of around 14.6 MeV energy created from DHe3 fusion reactions during exploding-pusher implosions. The target was a warm dense beryllium sample isochorically heated by multi-kilojoule laser-driven X-rays over few nanoseconds, reaching *T*_e_ ≈ 30 eV at solid density, corresponding to Γ ≈ 0.3 and Θ ≈ 2. However, as the beam-plasma interaction was in the high-velocity limit (*v*_p_/*v*_th_ ≈ 13), temperature and degeneracy effects on the stopping power were negligible, and the latter could be described by a simple Bethe-like formalism. Moreover, no detailed target characterization could be carried out, and only a small number of trials were able to be taken due to the scale of the laser facility. Meanwhile, lower-velocity regions (*v*_p_/*v*_th_ ≤ 10) in WDM have not been experimentally investigated until now, not to mention the Bragg peak region (*v*_*p*_ ∼ *v*_th_).

**Fig. 1:**
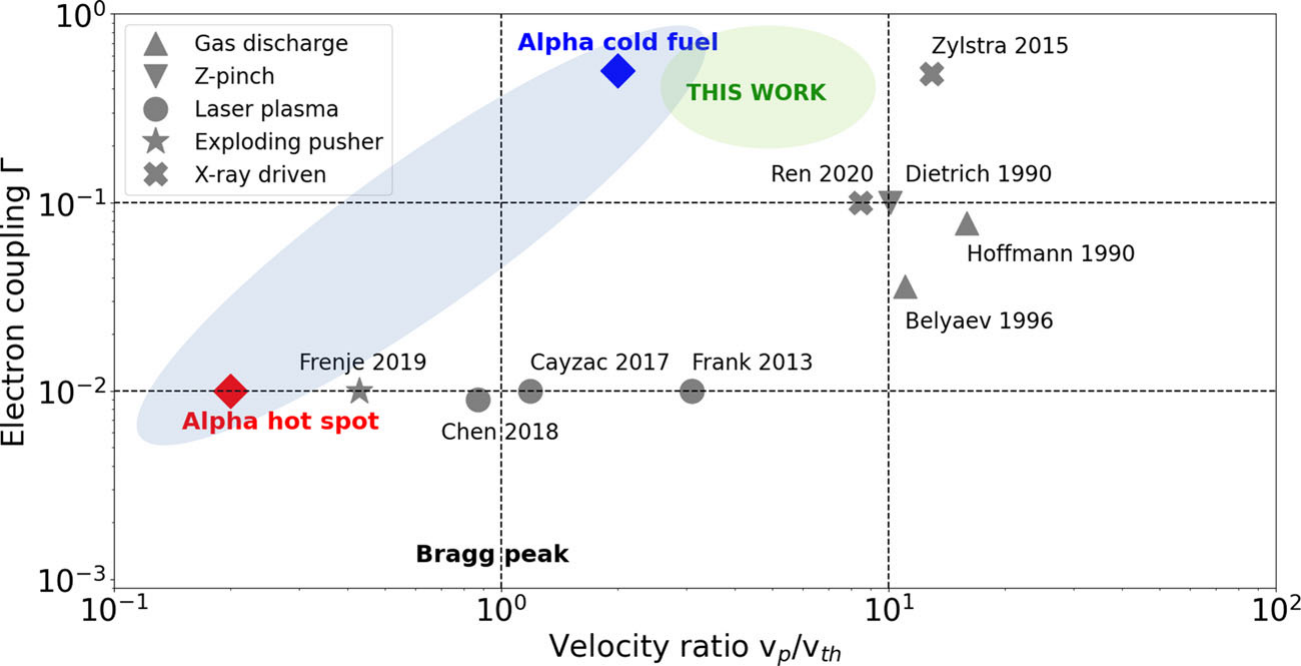
Selection of reported ion-stopping experiments. Experiments displayed in the parameter space of the velocity ratio *v*_p_/*v*_th_ of the beam-plasma interaction and the target electron coupling Γ. The grey symbols mark the plasma generation method used. The shaded blue zone represents the approximate range of *v*_p_/*v*_th_ and Γ values corresponding to the *α*-particle emission in an igniting ICF experiment, ranging from the cold fuel to the hot spot conditions. The experiment described in this work, indicated by the shaded green zone, lies in an unexplored parameter range that is relevant for *α*-particle stopping conditions in the cold fuel.

The parameter domain investigated so far is illustrated in Fig. 1, that shows a selection of reported stopping experiments displayed as a function of the velocity ratio *v*_p_/*v*_th_ and the electron coupling parameter Γ. Experiments performed in gas-discharge and Z-pinch targets^19–21^ are limited to low plasma densities (*n*_e_ ~ 10^17−18^ cm^−3^) and the high-energy probing ion beams on the MeV/u scale. Low to moderate velocity ratios *v*_p_/*v*_th_ ≤ 3 can be obtained in laser-generated plasma and exploding-pusher experiments, which are essentially limited to hot, ideal plasmas^22,25,26,35^. Cold and dense plasma conditions can be achieved with X-ray driven targets. While the plasma density remains ~10^20^ cm^−3^ in ref. ^36^, the experiment of ref. ^34^ does reach solid-density WDM conditions. However, the reported measurements involve high velocity ratios *v*_p_/*v*_th_ ≥ 10 due to the use of fast projectiles. Our goal is to simultaneously reach WDM states with moderate to strong electron coupling Γ ~ 0.1–1 and to probe them with low to moderate velocity ratio (*v*_p_/*v*_th_ ≪ 10), which remains an unexplored parameter domain approaching the conditions of *α* -particles in an ICF fuel shell and constitutes a step further towards the Bragg-peak region. Measurements at low velocity require well-characterized WDM samples and projectile ions with energies of a few hundred keV. Such low probing energies require thin samples which can experience a significant hydrodynamic expansion within tens to hundreds of picoseconds. Precise stopping measurements thus require a probing beam duration comparable or shorter than the sample lifetime. These requirements are difficult to achieve with accelerator ion beams because usual bunch durations lie on the nanosecond time scale. Exploding-pusher sources are limited by the relatively high (≥1 MeV) reachable projectile energies as well as proton pulse durations ~100 ps and by the availability of short-duration heater beams for the WDM sample generation. On the other hand, laser-generated proton beams, that feature short pulse lengths and broadband energy spectra, offer the required flexibility to overcome these limitations. Therefore, they have been used in several recent stopping experiments^35,36^ and are planned to be used in future experiments^37,38^, in general in association with an energy filtering device to select a narrow energy band. Moreover, as the stopping power at low velocity has a stronger temperature dependence, precise target temperature measurements are needed in order to both benchmark the plasma conditions and to interpret the energy-loss data.

In this work, we use an experimental approach based on a laser-generated proton selection platform operated at high-repetition rate at a short-pulse laser facility. Using this platform, we have measured the proton energy loss in a low-velocity regime in a warm dense carbon target that was heated by a second short-pulse laser. The projectile energy of around 500 keV led to velocity ratios *v*_p_/*v*_th_ between 3 and 10, significantly lower than in previous experiments (see Fig. 1). For these conditions, discrepancies between first-principles stopping-power calculations and classical predictions reach up to 20% and can be resolved experimentally. Our energy-loss measurements, in association with a detailed characterization of the WDM conditions using two complementary spectroscopy diagnostics, provide a first test of ion stopping models in this unexplored regime.

## Results

### Experimental setup

**Fig. 2:**
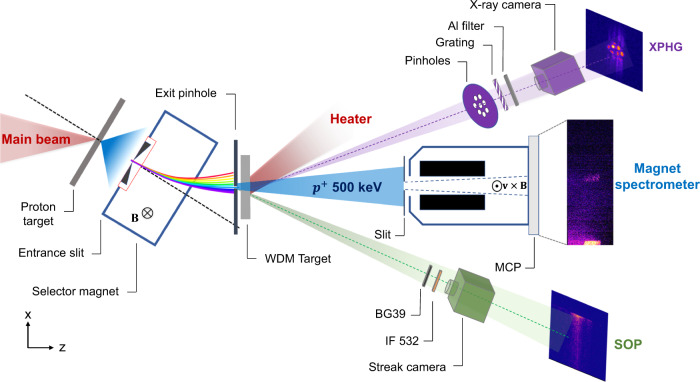
Experimental setup. Scheme of the experimental setup for each shot: (i) selection of a 500 keV energy proton beam from an initial broadband TNSA spectrum generated by the main beam, (ii) WDM sample generation by the heater beam, (iii) measurement of the downshifted proton energy spectrum of the selected beam after passing through the WDM target and (iv) characterization of the WDM sample by the SOP and the XPHG diagnostics. Typical raw experimental data acquired for each shot are shown for the magnet spectrometer as well as for the SOP and the XPHG diagnostics.

The experiment was performed at the PW-class VEGA laser facility at the Centro de Láseres Pulsados (CLPU), Salamanca, Spain^39^. The experimental setup is shown in Fig. 2. The initial 200 TW VEGA2 laser beam was split into two short pulses, respectively called the main and the heater beam. The setup consists of four main stages: (i) the generation of the proton beam by the main laser beam, (ii) the generation of the WDM sample by the heater beam, (iii) the measurement of the downshifted spectrum of the proton beam that passed through the WDM target using a magnet-based spectrometer and (iv) the characterization of the WDM conditions by using two independent spectroscopy diagnostics.

The main beam, with ≈ 4 J energy and a 30 fs duration, was focused onto a 3 μm thick aluminium foil in order to accelerate protons through the Target Normal Sheath Acceleration (TNSA) mechanism, resulting in a broadband spectrum^40^ with a cut-off energy around 4 MeV. A specifically developed magnetic filtering device^41^ was used to select a monoenergetic pencil-like proton beam of around 500 keV energy out of the initial spectrum to probe a target sample (solid or WDM state) located near the exit of the device. The proton beam diameter when entering the target was measured to be 50 μm using radiochromic films.

The WDM sample was generated by irradiating a carbon foil of 130 μg/cm^2^ initial areal density, corresponding to around 1 μm thickness, using the heater beam with a 0.5 J energy and an approximately 200 fs duration. The heater focal spot diameter was 300 μm, which is much larger than the proton beam spot size and maximizes the transversal uniformity of the target conditions probed by the proton beam.

The proton beam energy spectrum was measured at high repetition rate with a magnetic spectrometer coupled with a microchannel plate (MCP)^42^ featuring a resolution of 2 keV/pixel at 500 keV energy. In this way, the optimized selected proton beam was measured to have a 498 ± 4 keV central energy and a 44 ± 4 keV energy spread. The corresponding time spread when probing the target was estimated as 400 ± 15 ps by using the FLUKA Monte–Carlo code^43,44^. The experimental proton energy loss in the target was determined by the difference between the central energies of the selected proton beam spectrum measured after free propagation in vacuum and the downshifted proton beam spectrum measured after passing through the target.

Two independent spectroscopy diagnostics were employed to characterize the WDM conditions. A Streaked Optical Pyrometry (SOP) diagnostic^15^ was used to determine the time-resolved black-body WDM temperature within the area probed by the protons. It detected the optical emission from the heater side of the target at 532 nm wavelength with a temporal resolution of around 10 ps. Simultaneously, a XUV Pinhole Grating Camera (XPHG)^45,46^ measured the time-integrated X-ray emission in the XUV range from the WDM target heater side. The measurement was weighted over the whole heated area of around 500 μm diameter and had a spectral resolution of 9 nm.

### Simulations of the WDM target

**Fig. 3:**
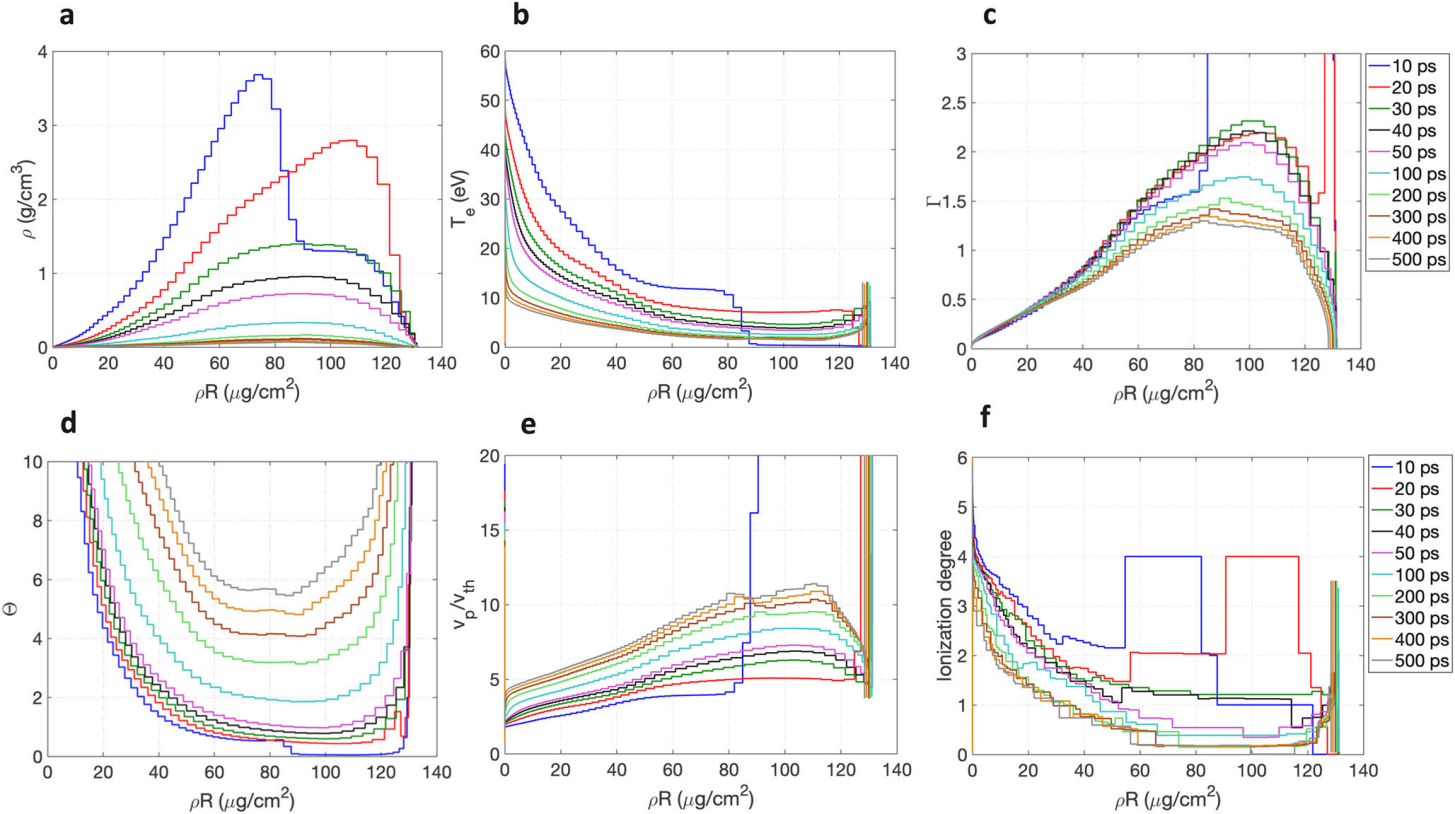
RALEF2D hydrodynamic simulation. Target profiles along the plasma central axis for *t* = 0–500 ps after the beginning of the laser heating. **a** Mass-density. **b** Electron temperature. **c** Electron coupling Γ. **d** Electron degeneracy Θ. **e** Velocity ratio *v*_p_/*v*_th_ for 500 keV energy projectiles. **f** Mean ionization calculated with the FLYCHK code at LTE. Discontinuities at early time are a calculation artefact. The *x*-axis is reported in areal-density units (μg/cm^2^). Sharp edges located at the target rear face (areal density ≈ 130 μg/cm^2^) are an isolated numerical simulation artefact.

The WDM conditions were simulated using the two-dimensional (2D) radiation-hydrodynamic code RALEF2D widely used for simulations of different experiments^47–49^, assuming local thermodynamic equilibrium (LTE), over a 500 ps time span after the heater beam onset on the target. The target ionization is deduced by post-processing the density and temperature profiles with the LTE version of the FLYCHK code^50^. The profiles of mass density *ρ*, electron temperature *T*_e_ and mean ionization Z^*^ along the target central axis are plotted in Fig. 3 for various times of the target evolution, where the target thickness is reported in areal-density units. The reached conditions are *ρ* ≥ 0.1 g/cm^3^ and *T*_e_ between a few eV and a few tens of eV, which correspond to carbon ionization degrees Z^*^ ≤ 4. The resulting values of Γ ≈ 0.1–2 and Θ ≤ 10 (in most of the target) are also shown in Fig. 3, indicating moderately to strongly coupled, and moderately degenerate target conditions. The velocity ratio values corresponding to the projectile energy of 500 keV are also plotted, with *v*_p_/*v*_th_ ≤ 10 over the considered time domain, and *v*_p_/*v*_th_ ≈ 2–3 in the first tens of picoseconds of the target evolution, which is significantly lower than in previous experiments. As also appears on the graphs of Fig. 3, the target areal density remains remarkably constant, which indicates a one-dimensional target expansion over the time range of 500 ps.

In addition, the interaction of the short-pulse heater beam with the target is likely to generate significant transient electric fields which may impact the proton beam and thus the energy-loss measurement. The effect of such fields was estimated by using a dynamic model of target charging by short-pulse laser interaction^51^. The target charging was estimated to dissipate within the first 10 ps after the heater beam onset on the target, which thus may influence only a small fraction of ≈ 2% of the beam protons and does not perturb the rest of the beam.

### WDM target characterization

**Fig. 4:**
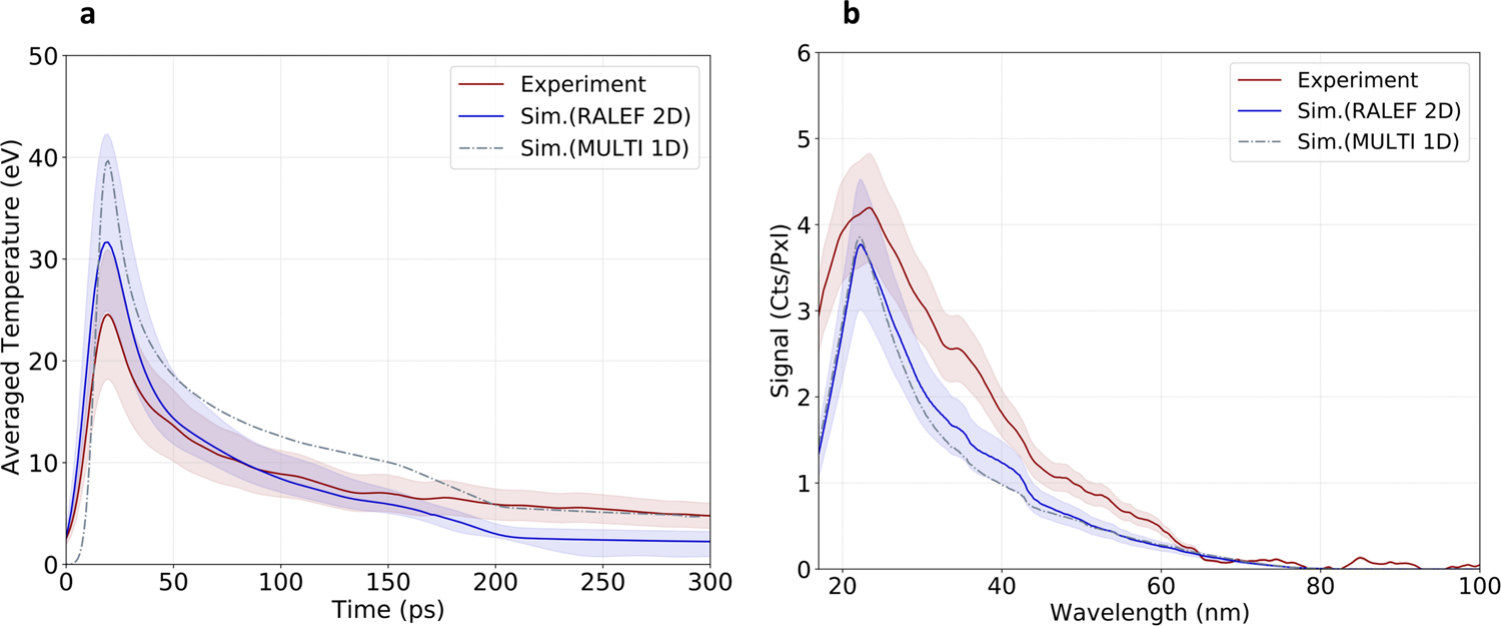
WDM Characterization. **a** Streaked Optical Pyrometry (SOP) measurement. Temperature evolution as a function of time (red curve) averaged within the 50 μm diameter proton probing area compared with the temperatures extracted from the 2D RALEF2D (blue curve) and the 1D MULTI-fs (dashed grey curve) hydrodynamic codes, determined at the critical density for a 532 nm wavelength. **b** X-ray pinhole grating camera (XPHG) measurement. Experimental time-integrated X-ray emission (red curve) compared with the prediction obtained with the PrismSPECT code by post-processing the hydrodynamic profiles obtained with the RALEF2D (blue curve) and MULTI-fs (dashed grey curve) hydrodynamic codes. The simulation curves are convoluted with the respective resolutions of 10 ps for the SOP diagnostic and 15 nm for the XPHG diagnostic.

The temporal evolution of the target temperature extracted from the SOP data averaged over 80 shots is shown in Fig. 4a and compared with the time-dependent temperature extracted from the RALEF2D simulation. Both the experimental and the simulated temperature are determined at the critical density for the 532 nm wavelength used for the measurements and averaged within a 50 μm emission diameter around the central plasma axis corresponding to the proton beam probing area. The experimental error results from the statistical error on the measurements and from the detector calibration uncertainty and is estimated as ±30%. The error band on the simulation curve accounts for the signal variation due to shot-to-shot pointing fluctuations of the heater beam estimated to be below 50 μm. The temperature determined from the SOP data is slightly lower than the one predicted by the RALEF2D simulation, while agreeing, in average, within the ±30% experimental error bar. The experimental temperature is also compared to the temperature extracted from a hydrodynamic simulation performed with the one-dimensional (1D) MULTI-fs code^52^ in LTE. The MULTI-fs prediction, also determined at the critical density for the 532 nm wavelength, overestimates the measured temperatures by around 30%. The RALEF2D prediction is clearly more accurate as the simulation was performed using the experimentally measured spatial distribution of the heater focal spot intensity.

The X-ray emission spectra over the whole target emission area measured with the XPHG diagnostic are presented in Fig. 4b. They are compared to the spatially and temporally integrated spectra obtained with the PrismSPECT code^53,54^ assuming LTE and using the density and temperature profiles extracted from both the RALEF2D simulation (Fig. 3a, b respectively) and from the MULTI-fs 1D hydrodynamic simulation carried out over a weighted range of intensities matching the experimentally measured focal spot. The measured spectra agree within 10–30% with the spectra predicted by the RALEF2D and the MULTI-fs codes, which is on the order of the experimental error bar estimated as ≈20%. In contrast to the SOP data, the XPHG measurement shows an X-ray emission at higher energies than simulated, which corresponds to an experimental temperature higher than simulated.

Based on the RALEF2D simulation, a mass-weighted and time-integrated temperature of 7.5 eV is estimated within the 50 μm proton diameter spot. Taking the average of both diagnostics, it can be concluded that the measured temperature is within 15% agreement with the RALEF2D simulation. The good agreement of the XPHG data with the RALEF2D prediction also shows that the target electron density is known with a reasonable accuracy. Moreover, the overall agreement of both measurements with the simulation over the whole considered time range indicates that the target expansion and thus the target areal density, are correctly simulated. In particular, the agreement of the experimental data with both the 2D and the 1D hydrodynamic simulations confirms that the target expansion is nearly one-dimensional as predicted by the RALEF2D code. Therefore, the presented spectroscopy measurement data set enables to validate the target parameters over the time domain of interest of a few hundred of ps. It is worth mentioning that this WDM sample characterization has been carried out simultaneously with the proton energy-loss measurements, which has not been done in previous stopping-power experiments.

### Stopping-power calculations

**Fig. 5:**
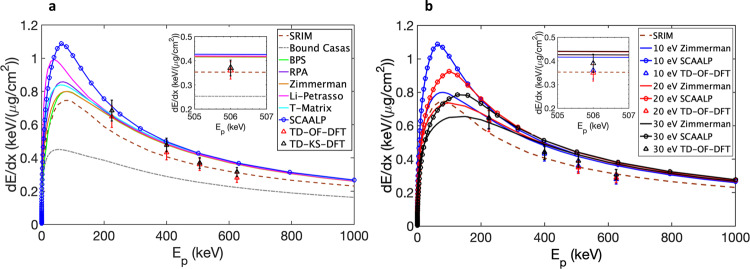
Comparisons of proton stopping power in warm dense carbon. **a** Stopping power for *ρ* = 0.5 g/cm^3^ and *T*_*e*_ = 10 eV. **b** Stopping power for *ρ* = 0.5 g/cm^3^ and various temperature values *T*_*e*_ = 10 eV, 20 eV and 30 eV. The error values of TD-OF-DFT and TD-KS-DFT results from systematic convergence studies of the box size and statistical variation of both the projectile pathways and finite stochastic vectors.

For estimating the discrepancies between stopping-power models for typical conditions of the experiment, various predictions for protons in carbon are compared in Fig. 5a for a density *ρ* = 0.5 g/cm^3^ and a temperature *T*_*e*_ = 10 eV. The proton stopping power in solid carbon according to the SRIM database^55^ is plotted as a reference.

Firstly, we use ad hoc calculations combining a free-electron and a bound-electron contribution that are obtained separately knowing the target ionization^56–58^. The free-electron term is calculated using several models that have the same Bethe-like high-velocity limit determined from dielectric stopping theory: the Li-Petrasso (LP) model^59^, the Brown-Preston-Singleton (BPS) model^60^, the T-matrix (TM) model with velocity-dependent screening^23,61^, the dielectric random phase approximation (RPA) model^23,62^ and the Zimmerman parametrization^63^ of the Maynard-Deutsch dielectric stopping power^64^, the latter being very similar to the RPA description. In all cases, the bound-electron stopping term, which is specifically plotted in Figs. 5a and b, is calculated using a model by Casas et al.^57^ that is valid for all projectile velocities.

Secondly, we use a self-consistent average-atom method in the local density approximation that simultaneously calculates the plasma ionization and the total stopping power using the method presented in refs. ^65,66^ and using the quantum average atomic model (QAAM) described in ref. ^67^ under the LTE assumption.

Thirdly, we employ an ab initio approach based on a recently developed time-dependent density functional theory (TD-DFT), including an orbital-free (TD-OF-DFT) version^30,31^ and a full Kohn–Sham approach (TD-KS-DFT) utilizing a mixed basis of deterministic and stochastic orbitals^68^. The target ionization in TD-DFT is defined self-consistently, as localized and delocalized electrons are naturally determined by the mean-field theory of DFT. Hence, there is no ad hoc separation between ionization and stopping-power physics. Moreover, the many-orbital representation of TD-KS-DFT allows for an exact treatment of Fermi-Dirac statistics, i.e. electron degeneracy, while TD-OF-DFT accounts for the degeneracy effects through a kinetic energy functional. It has been shown in refs. ^30,31^ that the TD-OF-DFT theory agrees with the high-velocity data of ref. ^34^, but predicts deviations of up to 20% from classical stopping-power predictions for WDM conditions at low projectile velocities. The TD-OF-DFT and TD-KS-DFT values are determined with an uncertainty estimated to ±10%.

The ad hoc and the QAAM calculations are in close agreement for proton energies *E*_p_ ≥ 500 keV and predict a significant increase of the stopping power compared to the solid, that reaches ≈ 20% at *E*_p_ = 500 keV. Discrepancies between these models increase at lower energies. In contrast, the TD-OF-DFT and the TD-KS-DFT theories predict a stopping power very close to the solid level for *E*_p_ ≥ 400–500 keV. The more precise TD-KS-DFT predictions are smaller and within better than 10% agreement with the TD-OF-DFT values. A stopping enhancement relative to the solid is also predicted but at lower velocities than according to other calculations, in the close vicinity of the Bragg peak. Hence, in the probing range of 500 keV energy, a 20% reduction of the stopping power is predicted by the TD-OF-DFT and TD-KS-DFT theories compared to the other models, which we attribute to the electron coupling and quantum degeneracy more precisely included in the TD-DFT calculations.

The effect of the target temperature on the stopping power is shown in Fig. 5b for an ad hoc calculation (namely with the Zimmerman model), and for the QAAM and TD-OF-DFT models, respectively, for the same density *ρ* = 0.5 g/cm^3^ and for temperatures *T*_e_ = 10, 20, and 30 eV. For these conditions, the ionization degree according to FLYCHK is Z^*^ = 1.43, 2.31 and 2.95, while the one estimated with QAAM is Z^*^ = 1.56, 2.40 and 2.86, respectively. As is shown, the stopping-power variation with temperature is very small for proton energies above 200–300 keV, reaching few percent at the experimental projectile energy of 500 keV. This also shows that the variation of the ionization degree with temperature on the one hand and the small ionization differences used for the various stopping-power calculations on the other hand are negligible for the studied conditions. These estimates thus suggest that the stopping power in the studied beam-target parameter range is not impacted by thermal effects, but that it is significantly impacted by coupling and/or degeneracy effects as shown by the discrepancy between the first-principle TD-OF-DFT calculation on the one hand and the classical and average-atom calculations on the other hand.

For comparison with the experimental measurements, we calculated the energy loss ${{\Delta }}{E}_{{{{{{{{\rm{sim}}}}}}}}}$ at each time step of the hydrodynamic simulation as the integral of the stopping power along the ion trajectory through the target

2 $${{\Delta }}{E}_{{{{{{{{\rm{sim}}}}}}}}}=-\int \frac{\partial E}{[\rho (x)\,\partial x]}[\rho (x)\,dx]\,,$$

where the stopping power, expressed as an energy loss per unit of areal density, is calculated with the parameter profiles as shown in Fig. 3. Each energy-loss value is averaged over the target parameters in a temporal range of 400 ps corresponding to the duration of the proton bunch interacting with the target, as well as in a spatial range of 50 μm corresponding to the probing proton beam diameter. The calculation was respectively performed for the cases where the proton beam is centered on the target central axis and where the proton beam is deviated by 50 μm from the central axis, which corresponds to the maximum estimated pointing fluctuation between the proton and the heater beam in the experiment.

For computational effectiveness, the energy loss is only calculated as follows. First, it is estimated in an ad hoc manner, using the Zimmerman, Li-Petrasso and T-Matrix models for the free-electron stopping and the Casas model for the bound-electron stopping. These three calculations predict very similar values within 1% (as also suggested by Fig. 5), and are simply designated as “classical calculation" in the following. Second, a TD-KS-DFT stopping-power fit is generated as a function of the target density and the projectile energy assuming a constant temperature *T*_e_ = 10 eV (see methods) for calculating a DFT-predicted energy loss.

The plasma parameters for our energy-loss calculation are well-characterized. Indeed, we neglect the experimental uncertainty of ±15% on the *T*_e_ measurement due to the low sensitivity of the stopping power to temperature in the studied conditions. Moreover, the uncertainty on the target areal density is negligible because of the 1D target expansion.

### Energy-loss results

Firstly, the energy loss of the proton beam was measured in solid carbon foils over 35 shots to estimate the measurement accuracy and provide a reference energy-loss value in the solid target Δ*E*_sol_. The downshifted proton energy after passing the solid target was measured to be 449 ± 5 keV, where the error *σ* = ± 5 keV results from the standard deviation at 1*σ* over all shots and from systematic measurement uncertainties as is explained in the methods. This results in an energy loss of Δ*E*_sol_ = 49 ± 5 keV, which is in good agreement with the energy loss of 48.1 keV predicted with the SRIM database^55^.

**Fig. 6:**
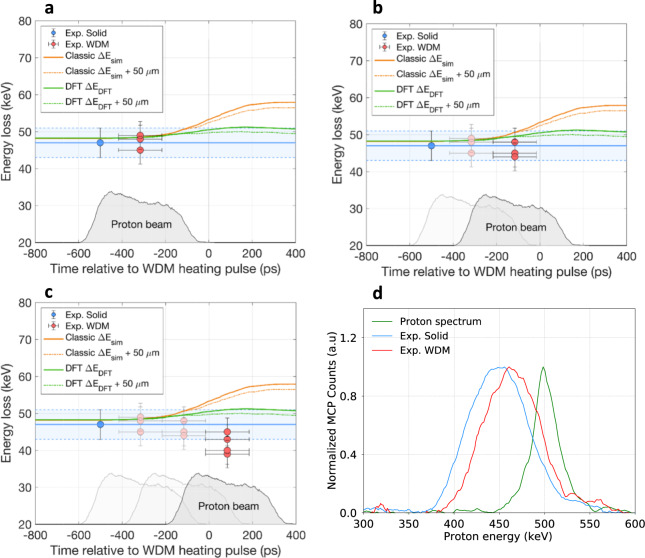
Experimental energy-loss results. Proton energy-loss data as a function of time. The time *t* = 0 ps corresponds to the onset of the heater beam on the carbon target. The temporal shape of the probing proton bunch obtained with a FLUKA Monte-Carlo simulation is represented as a shaded grey profile. The averaged measured energy loss in the solid target Δ*E*_sol_ is plotted as a blue solid line, and the surrounding light blue band indicates the error *σ* = ± 5 keV. The orange and green curves represent the results of the classical and of the KS-DFT energy-loss calculation assuming the proton beam probing the target along its central axis (solid lines) and with a 50 μm offset (dashed lines), respectively. **a** Energy-loss measurement at *t* = 316 ± 100 ps prior to the heater pulse onset. **b** Energy-loss measurement at *t* = 116 ± 100 ps prior to the heater pulse onset. **c** Energy-loss measurement at *t* = 86 ± 100 ps after the heater pulse onset. The vertical error bar is defined a quadratic sum of systematic and statistical error on proton energy measurement. The horizontal error bar comes from uncertainty of synchronization between heater and proton beam and it is defined as a quadratic sum of statistical error and uncertainty on proton time-of-flight estimation. **d** Comparison of the proton spectrum measured after free propagation in vacuum (averaged over 20 shots, green curve), the downshifted proton spectrum after passing the solid target (averaged over 35 shots, blue curve), and the downshifted proton spectrum after passing the WDM target (averaged over 4 shots, red curve). The vertical bars mark the spectra maxima positions.

Subsequently, the proton energy loss in the sample was measured on shots with the heater beam driving the target, at respective time delays of −316 ± 100 ps, −116 ± 100 ps and 86 ± 100 ps relative to the onset of the heater laser pulse on the sample. The experimental data acquired over several shots are presented at each time delay in Fig. 6a–c, where each data point corresponds to an individual shot. The blue band on each graph corresponds to the experimental error interval of ±5 keV of the reference energy-loss measurement in the solid target Δ*E*_sol_.

In Fig. 6a, b, the energy loss is measured before the laser heating of the sample, for protons still probing the solid target. The obtained data points are consistent with the previous reference energy-loss measurement in the solid foil Δ*E*_sol_.

In contrast, in Fig. 6c, the energy-loss measurement is performed when the temporal center of the proton beam is at 86 ps after the beginning of the sample heating, so that protons almost fully probe the WDM state. The measured energy loss reaches values between 36 ± 5 keV and 43 ± 5 keV depending on the shot, with an average value Δ*E*_WDMl_ of 39.4 ± 5 keV over four shots. This corresponds to values of 13–26% lower than the measurement in the solid target Δ*E*_sol_ of 49 keV ± 5 keV, with an average percentage difference of 20 ± 9%.

A comparison of the averaged proton spectra acquired respectively after free propagation in vacuum, after passing the solid target and after passing the WDM target is shown in Fig. 6d. The clear shift in the central proton energy between the spectra in the solid and in the WDM target indicates a reduced energy loss Δ*E*_WDMl_ in the WDM state.

The experimental data in Fig. 6a–c are compared with the results of the classical energy-loss calculation ${{\Delta }}{E}_{{{{{{{{\rm{sim}}}}}}}}}$. The calculation result assuming a 50 μm offset between the proton and the heater beam is slightly lower, by 1–2 keV, than the one along the central target axis due to lower temperatures of the probed region. At the time of proton probing in WDM, the calculated values ${{\Delta }}{E}_{{{{{{{{\rm{sim}}}}}}}}}$ are respectively 55.3 keV and 54 keV. These values are 15% and 12% higher than the SRIM energy loss in the solid target and 12% and 10% higher that the measured energy loss in the solid Δ*E*_sol_. Hence, the energy loss measured in the WDM sample, with an average value Δ*E*_WDMl_ = 39 ± 5 keV, is at least 15 keV lower than the classical prediction. These differences are greater than the error bars and thus suggest that the classical calculation ${{\Delta }}{E}_{{{{{{{{\rm{sim}}}}}}}}}$ overestimates the measured energy loss by 41%.

The experimental data are also compared with the results of the TD-KS-DFT energy-loss calculations Δ*E*_DFT_. The energy loss predicted at the time of proton probing in WDM Δ*E*_DFT_ = 51 ± 2.5 keV is only 6% higher than the energy loss measured in the solid target. Consequently, it overestimates the energy loss measured in WDM by 22.7 ± 14%. Therefore, the TD-KS-DFT calculations provide a twice better agreement with our experimental data than classical stopping-power models, which predict a much higher stopping-power enhancement for the considered WDM conditions and appear to be not valid in the probed parameter range.

In summary, our proton energy-loss data at 500 keV energy in warm dense carbon, at a velocity ratio down to *v*_p_/*v*_th_ ≥ 3, provide measurementt in the unexplored regime of low-velocity stopping in coupled and degenerate plasma conditions. When comparing these experimental measurements to existing stopping-power models, we find that the closest agreement is with the Density Functional Theory (TD-OF-DFT and TD-KS-DFT) calculations. This highlights the effect of electron coupling and degeneracy at low projectile energy in WDM, which reduces the stopping power compared to classical approaches. This result has strong implications for experiments where the energy loss of ions in WDM plays a significant role, where classical stopping-power modelling is usually employed. It thus calls for the use of more detailed calculations in this stopping regime based on first-principles methods like the Density Functional Theory. Moreover, our plasma emission measurements carried out using the SOP and XPHG spectroscopy diagnostics simultaneously to the stopping measurements, provide a WDM target characterization never achieved, to our knowledge, in previous stopping experiments. In particular, the experimentally determined temperature is in agreement within 20% with hydrodynamic simulations. This confirms the sample probing within the interesting regime of intermediate coupling (Γ ~ 1–2) and degeneracy (Θ ≤ 4). Ultimately, this work motivates further research in improved precision of the experimental measurements and development of theoretical models to narrow down the discrepancy.

In addition, the presented experimental platform is a promising tool for measuring the ion stopping power in the Bragg-peak region in WDM, where the largest theoretical discrepancies are reported. Our setup can span a large range of proton energies between 100 keV and 2 MeV as well as proton pulse duration and target temperature and density. For example, reducing the proton energy to 100 keV and keeping the same target temperature of ≈ 10 eV would enable to reach velocity ratios of *v*_p_/*v*_th_ ≈ 2, while increasing the target temperature to 20 eV would lead to even lower ratios of *v*_p_/*v*_th_ ≈ 1.3, achieving near-Bragg-peak conditions. Several developments of this experimental approach are possible by further refining the experimental parameters for increasing the precision and the accuracy of our measurements in order to provide more accurate comparisons with the theories. The proton energy selector can be optimized to reduce the proton beam bandwidth and time spread, with the goal of achieving a 5% measurement accuracy in the future. A proton focusing system can also be set up after the WDM target to mitigate the effects of angular straggling and maximize the proton collection, increasing the measurement precision by about 50%.

## Methods

### Experimental

#### Lasers and targets

The initial 4 J energy, 30 fs duration and 200 TW power VEGA 2 beam was split into two beams by using a 90% reflecting beam splitter^39^.

The main beam, that contains 90% of the total energy, was used to accelerate protons via the Target Normal Sheath Acceleration (TNSA) mechanism. It was focused using an *F*/13 (*F* = 130 cm) parabolic mirror onto a 3 μm thick aluminium foil at a 14.5^∘^ incidence angle. The pulse duration was 30 fs and the focal spot diameter was 20 μm at full width at half maximum (FWHM), yielding an intensity on target of ~ 10^19^ W/cm^2^. The aluminium foils had dimensions of ≈ 80 × 80 mm and were mounted in a motorized sandwich holder with a matrix of 45 × 45 apertures of 800 μm diameter each. Each aperture was used for one shot, which allowed a quick shot-to-shot target switch for operation at high repetition rate.

The heater beam containing the remaining 10% laser energy was stretched to a 217 fs pulse duration that was measured using a second-order autocorrelator with an accuracy of 5 fs. For generating the WDM sample, the beam was focused onto a carbon target with an incidence angle of 35^∘^ and a focal spot diameter of 300 μm, which yielded an intensity on target ~10^16^ W/cm^2^. The carbon target was positioned at a 0.9 cm distance from the exit pinhole of the selector in the proton propagation axis and at a ~ 8 cm distance from the proton source point.

The targets were portions of large 80 mm diameter carbon foils that were manufactured by resistance evaporation under high vacuum^69^ at the GSI Target Laboratory. The energy-loss measurements in the solid and in the WDM target presented in this work were performed with the same carbon foil with an average initial areal density of 130 μg/cm^2^ with an uncertainty of ±1%. With a carbon density ≈1.3 g/cm^3^, this areal density value corresponds to a 1 μm initial target thickness. For allowing measurements at high repetition rate, the foils were mounted in a holder with a matrix of 45 × 45 apertures, each aperture corresponding to one target used on one shot. The aperture diameter was of 1 mm on the proton incidence side and of 800 μm on the heater-beam side, which was designed to ensure the integrity of neighbouring targets during each shot because of the high fragility of the foil. The good agreement of the measurements in solid carbon with the SRIM prediction for a 130 μg/cm^2^ areal density shows that possible effects of areal-density variations across the foil surface are within the experimental energy-loss error bars and do not impact the data analysis.

#### Magnet spectrometer

The magnet spectrometer was designed and characterized at CLPU and was used to measure the proton beam energy. It was positioned at a 38.2 cm distance from the WDM sample along the proton propagation axis. The spectrometer consists of a 0.2 T, 10.4 cm long dipole magnet. It deflects protons upwards to a microchannel plate (MCP) detector, which is coupled with a phosphor screen located 10 cm from the end of the magnet and imaged onto a CCD camera. The 2D magnetic field of the spectrometer was measured with a Hall effect probe and was used to calculate the predicted proton deflection on the MCP. The resolution of the spectrometer at 500 keV proton energy is 2 keV per pixel of the image. A horizontal slit of 1 mm height and 1 cm length was inserted in front of the spectrometer entrance aperture of 1 cm diameter to ensure that only protons within the horizontal plane of the propagation axis enter the spectrometer. This provides the “zero height" (zero deflection) reference position on the detector. The vertical positioning uncertainty of the proton beam of ±125 μm results in an energy uncertainty of ±2.5 keV on the MCP that constitutes a systematic error ${\sigma }_{{{{{{{{{\rm{sys}}}}}}}}}_{1}}$ on the energy measurement.

**Fig. 7:**
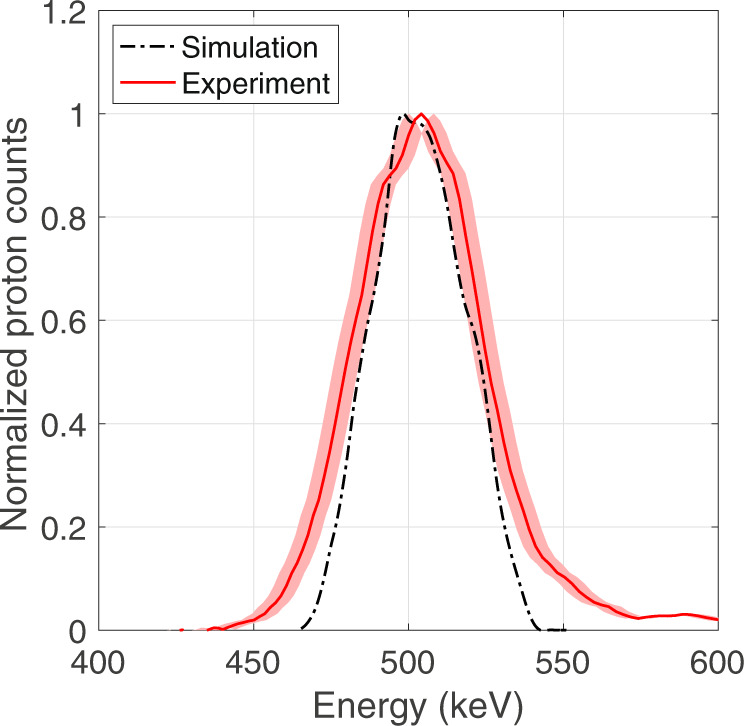
Selected proton beam spectrum. Comparison between the measured selected proton beam spectrum (red solid curve) and the selected proton beam spectrum obtained from an initial broadband TNSA-like spectrum with energies of 0 – 2 MeV simulated with the FLUKA Monte-Carlo code using the experimental configuration for the proton energy selector (black dashed curve). The shaded area around the red curve represents the measurement error.

Examples of raw images obtained with the MCP detector for individual shots are presented in Fig. 7, which shows a reference signal of the selected proton beam (a) and selected proton beam signals after passing through target samples (b–d). As is visible in Fig. 7b, the angular straggling of the proton beam through the target, which is estimated to be around 2^∘^ using FLUKA simulations, results in a 3 cm spot at the spectrometer entrance. This signal broadening introduces an error in the estimation of the central energy of the downshifted proton spectrum. In order to mitigate this error, we mounted a horizontal slit of 1 mm height in front of the spectrometer for reducing the beam spot size on the MCP detector as illustrated in Fig. 7c. Using this slit, a systematic error is added on the energy measurement in the sample due to partial collection of protons on the detector, which is estimated as ${\sigma }_{{{{{{{{{\rm{sys}}}}}}}}}_{2}}=\pm 3.5$ keV.

The total error on the energy-loss measurement is estimated as ${\sigma }_{{{{{{{{\rm{tot}}}}}}}}}=\sqrt{{\sigma }_{{{{{{{{\rm{stat}}}}}}}}}^{2}+{\sigma }_{{{{{{{{{\rm{sys}}}}}}}}}_{1}}^{2}+{\sigma }_{{{{{{{{{\rm{sys}}}}}}}}}_{2}}^{2}}$, where ${\sigma }_{{{{{{{{\rm{stat}}}}}}}}}^{2}={\sigma }^{2}/N$. Here, *σ* is the standard deviation and *N* is the number of shots, while ${\sigma }_{{{{{{{{{\rm{sys}}}}}}}}}_{1}}=\pm$2.5 keV and ${\sigma }_{{{{{{{{{\rm{sys}}}}}}}}}_{2}}=\pm\!3.5$ keV are the systematic errors coming respectively from the proton beam alignment and from the aforementioned partial collection of protons. The energy loss in the target is estimated as the difference between the measured central energies of the reference and of the downshifted beam, and can be written as ${{\Delta }}{E}_{{{{{{{{\rm{down}}}}}}}}}={E}_{{{{{{{{\rm{ref}}}}}}}}}({\sigma }_{{{{{{{{\rm{stat}}}}}}}}},{\sigma }_{{{{{{{{{\rm{sys}}}}}}}}}_{1}})-{E}_{{{{{{{{\rm{down}}}}}}}}}({\sigma }_{{{{{{{{\rm{stat}}}}}}}}},{\sigma }_{{{{{{{{{\rm{sys}}}}}}}}}_{1}},{\sigma }_{{{{{{{{{\rm{sys}}}}}}}}}_{2}})$.

#### Energy selector

The energy selector was designed and characterized at CLPU as a compact adjustable platform for proton stopping-power measurements with working range energies of up to a few MeV. It is ~6 cm long and it consists of a 1.2 T permanent dipole magnet that deflects protons in the horizontal plane using two apertures. The first one is placed at the magnet entrance (entrance slit) and the second one at the magnet exit (exit pinhole). The selector is positioned at 1.6 cm from the proton source and it is rotated by 14.5^º^ for pointing the selected proton beam in straight axis with respect to the WDM sample. The entrance slit, of 20 μm width and 3 mm height, is attached in front of the dipole magnet yoke for reducing the horizontal acceptance of the incoming TNSA proton beam. The selected pencil-like proton beam undergoes a horizontal energy spread after entering into the magnetic field region. The exit pinhole of 20 μm diameter, positioned at 1 cm after the exit of the magnet, selects a narrow bandwidth of proton beam energies that freely propagates up to the carbon sample. The selector is designed to be fully operational at high repetition rate with a motorization of the dipole magnet moving in and out and a holder for the exit pinhole with horizontal and vertical motorization. The design and the optimization of the energy selector are presented in detail in ref. ^41^. In this work, we selected a proton beam with a central energy of 498 ± 4 keV and an energy bandwidth of 44 ± 4 keV at FWHM, where 4 keV is the total uncertainty for a single shot. The selected proton energy was found to be highly reproducible from shot to shot within a statistical error of ±2.6 keV (given by ${\sigma}_{{{{{{{{\rm{stat}}}}}}}}}=\sigma /\sqrt{N}$, where *σ* = 12 keV is the measurement standard deviation and *N* = 20 is the shot number). Such small error also suggests a low sensitivity of the selected proton beam parameters to the laser shot-to-shot instability (pointing stability of ~ 12 μm, energy variation ~ 3%).

**Fig. 8:**
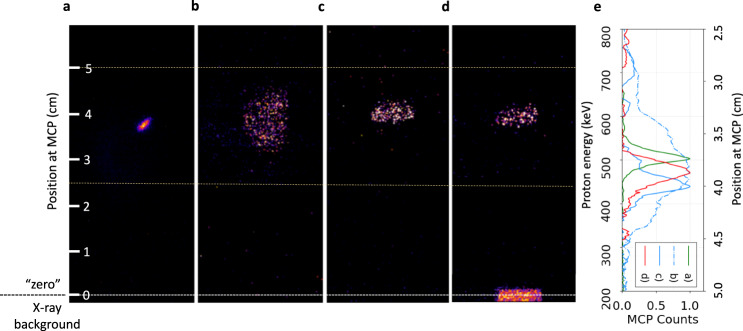
Example of raw data acquired with the MCP detector coupled to the magnet spectrometer. The total height of the MCP phosphor screen is 7.8 cm. Each image shows the acquisition from an individual shot, with a voltage of 5000 V and a gain of 20. The “zero" point that corresponds to the “non deflected" reference position of the proton beam is found by locating the center of the X-ray background along the vertical direction as indicated by the dashed white line. **a** Selected proton beam signal obtained with a 20 μm entrance slit and a 20 μm exit pinhole on the energy selector. **b** Selected proton beam signal after passing through the solid carbon foil. **c** Selected proton beam signal after passing through the solid carbon foil with a 1 mm horizontal slit inserted in front of the spectrometer entrance. **d** Selected proton beam signal after passing through the WDM sample with a 1 mm horizontal slit inserted in front of the spectrometer. The X-ray background is produced by the heater beam interaction with the target. **e** Vertical lineouts of the images (**a**), (**b**), (**c**), (**d**) as a function of the position on the MCP screen and of the corresponding energy calculated using the energy dispersion by the magnetic field.

The energy spectrum of the selected proton beam measured with the high-resolution magnet spectrometer is shown in Fig. 8 and compared with a synthetic spectrum obtained with a FLUKA Monte-Carlo simulation^43,44^ using the experimental selector and detector geometry. The experimental data and the simulated spectrum are in good agreement in their widths at FWHM, while slight differences appear at the wings of the spectra due to proton beam divergence. In addition, the experimental design of the selector ensures that only protons are present at the probing time of the WDM sample. If there were any carbon ions generated by the TNSA process (typically C^4+^ and C^3+^), they would interact with the WDM sample more than 9 ns later than the proton beam and thus would have no effect. Moreover, the 2 μm thick mylar foil placed in front of the MCP would prevent any carbon ions from reaching the MCP and from being detected.

#### Synchronization between the proton and the heater beam

The sub-ns time synchronization was performed for both laser beams at their respective interaction points (proton target and WDM target position) accounting for the time-of-flight (TOF) for 500 keV protons between these points. The proton trajectory was calculated analytically based on the experimental geometry and verified using Monte–Carlo simulations. The heater beam was delayed in respect to the main beam by the proton TOF of 9.2 ns up to the WDM sample position. This was achieved with the help of a 3 m long delay line for increasing the heater beam path. This main delay line was coupled with a smaller motorized delay line of 20 cm length enabling a fine adjustment with a minimum time step of 10 ps. The main and the heater beams were synchronized with a 9.2 ns delay by using photodiodes positioned at their respective interaction points. Both pulse signals were adjusted on a 1 GHz oscilloscope with identical cable lengths using the smaller delay line. The required delay value was obtained with a precision of ±100 ps, calculated as ${\sigma }_{{{{{{{{\rm{tot}}}}}}}}}=\sqrt{{\sigma }_{{{\rm{stat}}}}^{2}+{\sigma }_{{{{{{{{\rm{p}}}}}}}}}^{2}}$, where *σ*_stat_ = 70 ps is a statistical error and *σ*_p_ = 50 ps is the error on the proton TOF calculation.

#### XUV pinhole grating (XPHG) diagnostic

The XPHG diagnostic, based on a a free-standing multi-pinhole X-ray transmission grating, was used to measure the broadband XUV emission from the plasma^45,46^. It had a target view of 30° in relation to the target normal on the heater beam side. The 500 line/mm grating consisted of gold bars of 1 μm width and thickness with 1 μm openings. It had a larger reinforcement grid structure resulting in an open grating area of 80%, as measured with a scanning electron microscope image. The diffraction efficiency into each of the plus and minus first-order spectra was taken as 1/*π*^2^ = 0.101, which is the ideal response for such a transmission grating, as was verified by previous authors for this wavelength range^70^. The grating had multiple 400 μm pinhole openings and the spectra were obtained from two of the pinholes. The spectra presented are the weighted average of three such single sided spectra per laser shot and averaged over the order of 50 laser shots per experiment. The spectra were filtered by a 400 nm thick aluminium foil, which transmitted X-rays from 17 nm to around 70 nm wavelength, and detected using an Andor iKon-M XUV CCD camera. The distance from the plasma to the pinhole grating was 1197 mm and the distance from the pinhole to the CCD camera was 99 mm. The diagnostic dispersion was calculated using the distance to the camera, the pixel size and the grating spacing, with an estimated overall accuracy on the order of ±3%. The camera response in counts per keV deposited has been absolutely calibrated with single photons at 5.9 keV energy from an Fe-55 radioisotope X-ray source. The relative response at the XUV wavelengths of interest of 17 nm to 70 nm was taken from the manufacturer’s published spectral response curve for the camera. Taking the geometric factors, the transmission factors and the response function of the camera into account, absolute emission values were obtained. The estimated accuracy of the absolute emission measurement is on the order of ±20%.

#### Streaked optical pyrometry (SOP) diagnostic

The Streaked Optical Pyrometry (SOP) diagnostic was used for measuring the time-resolved black-body temperature of the WDM target with a 10 ps resolution. Due to its sensitivity to low temperatures, this diagnostic is well-suited for measuring the temperature of WDM samples^15^. The SOP diagnostic had a target view of 25° in relation to the target normal on the heater beam side.

The emission of the WDM target was collected by the optical system, imaging a region of ~ 400 μm onto a Hamamatsu S20 streak camera with a magnification of 5. The interferometric filter was centered at a 532 ± 0.6 nm wavelength with a FWHM bandwidth of 3 ± 0.6 nm (FI532). An additional color-glass bandpass filter for wavelengths of 360–580 nm (BG39) was used to mitigate the laser light at 800 nm wavelength propagating along the collection axis. The wavelength-dependent response of the SOP system within the 3 nm bandwidth was provided by the manufacturers. The transmission of the optical system for SOP has been measured with a 532 nm continuous diode laser and the streak camera was absolutely calibrated at the selected wavelength using the calibration of ref. ^71^. The latter was carried out with the same streak camera employed in this experiment and with the filter data set BG38 and FI532 that are similar to the ones we used. The data were acquired inside a time window of 2 ns. The temporal evolution of the temperature was determined using a vertical line-out of the central target region of 50 μm diameter corresponding to the proton beam size entering the WDM target. The resulting experimental curve averaged over 65 shots is presented in Fig. 4. The error bar is estimated as ${\sigma }_{{{{{{{{\rm{total}}}}}}}}}=\sqrt{{\sigma }_{{{{{{{{\rm{SE}}}}}}}}}^{2}+{\sigma }_{{{{{{{{\rm{stat.}}}}}}}}}^{2}+{\sigma }_{{{{{{{{\rm{calib.}}}}}}}}}^{2}}$ that includes the standard error ${\sigma }_{{{{{{{{\rm{SE}}}}}}}}}=\sigma /\sqrt{N}$ where *σ* is the standard deviation from the mean, a 25% statistical error *σ*_stat._ and a 5% uncertainty in the detector calibration *σ*_calib._.

### Modelling

#### RALEF2D and MULTI-fs hydrodynamic simulations

The RALEF2D simulation was performed in axi-symmetric geometry using the experimentally measured laser parameters, namely an energy of 0.45 J, a gaussian-shaped temporal laser pulse profile with a 217 fs width at FWHM and a spatial distribution profile of the focal spot of ≈ 150 μm radius.

The density and temperature profiles are sampled with a 5 ps time step for *t* = 0–100 ps and a 10 ps step for *t* = 110–500 ps. The spatial sampling is of ≈ 350 points for longitudinal rays (along the proton propagation axis) over the target areal density, and of 5 μm in the transverse direction up to a radius of 150 μm.

The MULTI-fs 1D simulation was performed using the same laser energy and pulse duration. In order to represent the radial intensity profile of the focal spot, four separate simulations were performed using the input intensity of the heater calculated within an effective radius that contains 7, 20, 50, 90% of the total laser energy. The input intensities were used as following: *I*_1_ = 7.4 × 10^15^ W/cm^2^ at 25 μm effective focal spot radius containing 7% of energy, *I*_2_ = 5.2 × 10^15^ W/cm^2^ at 50 μm focal spot containing 20% of energy, *I*_3_ = 1.85 × 10^15^ W/cm^2^ at 133.5 μm containing 50% of energy and *I*_4_ = 7.39 × 10^14^ W/cm^2^ at 283.9 μm containing 90% of energy. For the precision of the calculation of the hydrodynamic parameter values, the target was sampled into 200 layers. The density and temperature profiles were obtained at each layer of the target and sampled with a 10 ps step for *t* = 0–300 ps.

#### Proton energy-loss calculations

The energy-loss simulations are performed similarly as in ref. ^24^. The ionization distribution of the plasma is calculated using the collisional-radiative FLYCHK code in local thermal equilibrium^50^, which provides the ion densities (*n*_0_, …, *n*_6_) of the different plasma charge states (C^0+^, …, C^6+^) for each point of the considered profile. The free electron density is calculated as *n*_e_ = 6 *n*_6_ + 5 *n*_5_ + 4 *n*_4_ + 3 *n*_3_ + 2 *n*_2_ + *n*_1_. The mean plasma ionization degree Z^*^ is then determined from the relation *n*_e_ = Z^*^ *n*_*i*_. Here, *n*_i_ = *ρ* *N*_A_/*A*_t_ is the total ion density, where *A*_t_ = 12 is the molar mass of carbon and *N*_*A*_ is the Avogadro number. The free-electron stopping power is calculated using the density *n*_e_ with the Zimmerman, Li-Petrasso and T-Matrix models. The bound-electron stopping power is determined using the ion densities *n*_0_, …, *n*_5_ and the Casas model. The carbon atomic quantities required for the bound electron calculation are taken from ref. ^57^. The total stopping power is obtained as the sum of these contributions:

3 $${\frac{dE}{dx}}_{{{{{{{{\rm{total}}}}}}}}}={\frac{dE}{dx}}_{{{{{{{{\rm{free}}}}}}}}}+{\frac{dE}{dx}}_{{{{{{{{\rm{bound}}}}}}}}}$$

**Fig. 9:**
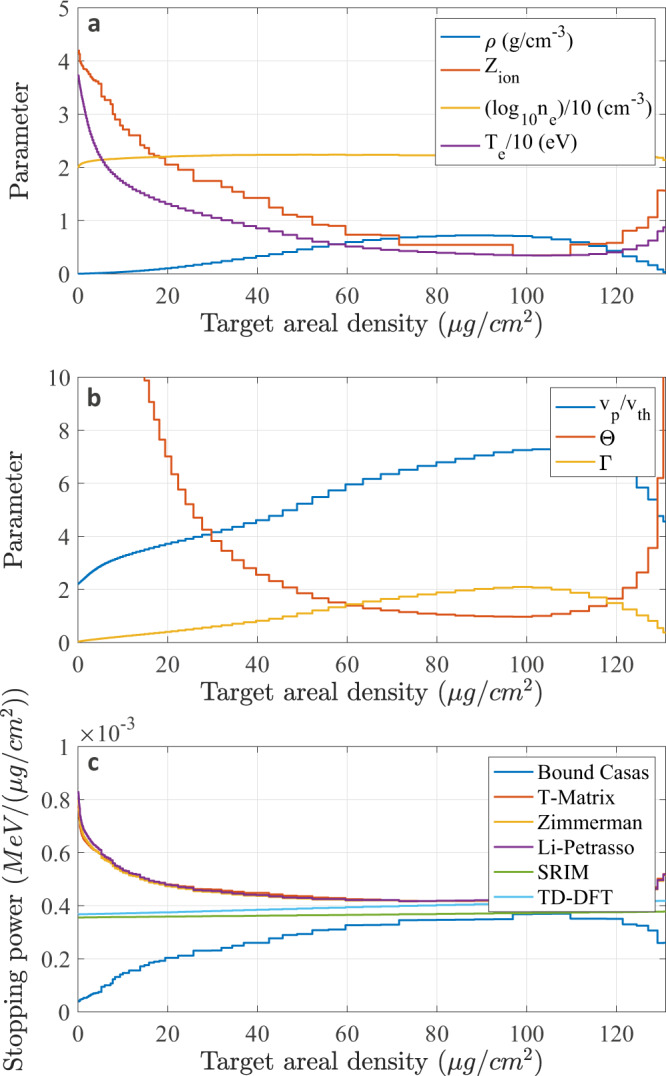
Plasma conditions and stopping power along the plasma central axis at a time *t* = 50 ps. **a** Mass-density, electron temperature, free electron density and mean ionization degree. **b** Electron coupling Γ, electron degeneracy Θ and velocity ratio *v*_p_/*v*_th_ for a 500 keV energy proton. **c** Corresponding stopping-power profiles. The bound-electron contribution as well as the stopping power in the solid target according to the SRIM database are also represented. The *x*-axis is reported in areal-density units (μg/cm^2^).

The projectile charge state is modeled using the effective charge state predicted by Gus’kov et al.^72^, which is valid in plasma at any projectile velocity. At 500 keV projectile energy, it reaches values ≈ 0.98–0.99 depending on the target conditions. The projectile slowing down inside the target is taken into account for each step along the proton propagation path for the beam charge state and the stopping-power calculation. An illustration of stopping-power profile calculation is shown in Fig. 9 for the plasma conditions along the target central axis at *t* = 50 ps after the beginning of the laser target heating. The target density, temperature, ionization and free electron density profiles are shown in Fig. 9a, the corresponding Γ, Θ and *v*_p_/*v*_th_ values are shown in Fig. 9b, and the resulting stopping power for one proton of initially 500 keV energy is shown in Fig. 9c. The three ad hoc calculations provide almost identical results, consistently with Fig. 5. Meanwhile, the result of our KS-DFT fit (which is explained below) lies between the SRIM curve and the classical values. The energy loss for one proton at this time step is obtained as the integral of the represented stopping power. The energy loss values obtained for all time steps are then convoluted with the spatial and temporal profiles of the probing proton beam. For this purpose, a Monte–Carlo calculation is performed assuming a beam energy bandwidth of 44 keV at FWHM, i.e. a temporal width ≈ 400 ps at FWHM, and a spatial width of 50 μm at FWHM. The simulation is performed with the approximate estimated proton number per bunch of 1000.

#### TD-DFT stopping-power calculations

The time-dependent orbital-free density functional theory (TD-OF-DFT)^30,31^ formulation included a nonadiabatic, temperature-dependent kinetic-energy density functional and an exchange-correlation contribution in a local density approximation as well as the usual Hartree and external terms with a local all-electron pseudopotential for carbon. Rectangular prisms of 512 atoms with dimensions 70.0 × 17.5 × 17.5 Å were employed as reference cells with the atomic configurations determined from an equilibrium orbital-free molecular dynamics simulation. For a given projectile velocity, the total electron stopping power is determined by the work on the proton as a function of the distance travelled averaged over 2–3 atomic configurations, 10–15 initial positions for the proton, and 3–4 passages of the proton through the cell. For the 10 eV case, TD-OF-DFT calculations with 2048 atoms and 70 × 35 × 35 Å cells were also performed to conclude that finite size effects were not a significant factor at the reported projectile velocities.

TD-KS-DFT calculations for the WDM case were performed using 256 atoms in a 17.5 × 17.5 × 35 Å cell with an energy cutoff of 1035 eV and the PZCA LDA exchange correlation functional^74^. 18 trajectories per velocity point were used to average the stopping power. A mixed stochastic-deterministic representation of the Kohn–Sham orbitals was employed, with 16 deterministic and 64 stochastic orbitals. Further details can be found in ref. ^73^. TD-KS-DFT calculations for the amorphous solid were performed using 256 atoms in a 9.9 × 9.9 × 39.7 Å cell with an energy cutoff of 1111 eV and the PBE GGA exchange correlation functional^75^.

The TD-KS-DFT stopping power has been calculated for 400, 506 and 624 keV projectile energies and plasma densities of 0.5 g/cc, 3.5 g/cc and 10 g/cc, respectively. Due to the high computational cost, only 10, 10 and 5 trajectories were used for the 400, 506 and 624 keV energy proton calculations respectively. 520 deterministic Kohn-Sham orbitals were used in each case. The electron temperature is assumed fixed to 10 eV, which is representative of the target conditions and takes advantage of the low sensitivity of the DFT stopping power to the temperature variation as shown in Fig. 5b. These simulations were used to generate polynomial fits as a function of target density and projectile energy which were implemented in our energy-loss calculation routine.

The target ionization Z^*^ is treated as follows. Classical models utilize isolated atom wavefunctions with Casas’ approach^57^ for bound electrons 6-Z^*^ electrons per atom, with plasmas models based on a homogeneous electron gas for the Z^*^ remaining electrons. In contrast, DFT utilizes the same Casas method for the 1*s* electrons, which are implicitly treated in the DFT calculations by pseudopotentials, but treats the remaining 4 electrons per carbon in a full multi-atom, i.e. atomistic, calculation. Thus, delocalized electrons are subject to the full disordered many-atom ion potential, which is critical in this density regime where some electrons are indeed 2*s* and 2*p*-like, while others are highly delocalized^73^.

#### XPHG and SOP diagnostic modelling

For the comparison with the experimental XUV spectra obtained with the XPHG diagnostic, the PrismSPECT atomic code was used to postprocess target profiles extracted from the RALEF2D and MULTI-fs 1D hydrodynamic simulations. Using the RALEF2D simulation, we considered target profiles at radii *r* = 30, 60, 90, 120, 150 μm from the proton propagation axis (target center), over the expansion time of 0–300 ps, with time steps of 10 ps for *t* = 0–100 ps, and 50 ps for *t* = 100–300 ps. At each time step, the spatially-integrated emission spectrum was obtained by summing the area-weighted emissivity at each radius. The spectra were then integrated over time to obtain the total space- and time-integrated emission as measured by the XPHG diagnostic on the heater beam side of the target. As for the MULTI-fs simulation, we used target profiles of each of the four simulations with different heater intensities over the expansion time of 0–300 ps, with time steps of 10 ps for *t* = 0–100 ps, and 50 ps for *t* = 100–300 ps to calculate the emissivity with PrismSPECT. For obtaining an area-weighted emissivity at each time-step, a simple model was employed to numerically determine a radius for each average ring of intensity used in simulations and calculate the area.

In order to compare the experimental temperature temporal evolution obtained with Streaked Optical Pyrometry, we considered the RALEF-2D expansion profiles averaged over the central 50 μm diameter area and the MULTI-fs expansion profiles of the simulation with the intensity that corresponds to the effective proton probe spot radius of 25 μm. For this purpose, we considered the temperature with a time step of 10 ps at the critical density *n*_*c*_ = 3.88 × 10^21^ cm^−3^ that corresponds to the wavelength *λ* = 532 nm.

## Data Availability

The datasets generated during and/or analyzed during the current study are available from the corresponding author on reasonable request.
